# Risk assessment for long- and short-range airborne transmission of SARS-CoV-2, indoors and outdoors

**DOI:** 10.1093/pnasnexus/pgac223

**Published:** 2022-10-06

**Authors:** Florian Poydenot, Ismael Abdourahamane, Elsa Caplain, Samuel Der, Jacques Haiech, Antoine Jallon, Inés Khoutami, Amir Loucif, Emil Marinov, Bruno Andreotti

**Affiliations:** Laboratoire de Physique de l’Ecole Normale Supérieure (LPENS), CNRS UMR 8023, Ecole Normale Supérieure, Université PSL, Sorbonne Université, and Université Paris Cité, 24 rue Lhomond, 75005 Paris, France; Laboratoire de Physique de l’Ecole Normale Supérieure (LPENS), CNRS UMR 8023, Ecole Normale Supérieure, Université PSL, Sorbonne Université, and Université Paris Cité, 24 rue Lhomond, 75005 Paris, France; Laboratoire de Physique de l’Ecole Normale Supérieure (LPENS), CNRS UMR 8023, Ecole Normale Supérieure, Université PSL, Sorbonne Université, and Université Paris Cité, 24 rue Lhomond, 75005 Paris, France; Laboratoire de Physique de l’Ecole Normale Supérieure (LPENS), CNRS UMR 8023, Ecole Normale Supérieure, Université PSL, Sorbonne Université, and Université Paris Cité, 24 rue Lhomond, 75005 Paris, France; Cogitamus Laboratory and CNRS UMR 7242 BSC, 300 Bd Sébastien Brant, CS 10413, 67412 Illkirch Cedex, France; Laboratoire de Physique de l’Ecole Normale Supérieure (LPENS), CNRS UMR 8023, Ecole Normale Supérieure, Université PSL, Sorbonne Université, and Université Paris Cité, 24 rue Lhomond, 75005 Paris, France; Laboratoire de Physique de l’Ecole Normale Supérieure (LPENS), CNRS UMR 8023, Ecole Normale Supérieure, Université PSL, Sorbonne Université, and Université Paris Cité, 24 rue Lhomond, 75005 Paris, France; Laboratoire de Physique de l’Ecole Normale Supérieure (LPENS), CNRS UMR 8023, Ecole Normale Supérieure, Université PSL, Sorbonne Université, and Université Paris Cité, 24 rue Lhomond, 75005 Paris, France; Laboratoire de Physique de l’Ecole Normale Supérieure (LPENS), CNRS UMR 8023, Ecole Normale Supérieure, Université PSL, Sorbonne Université, and Université Paris Cité, 24 rue Lhomond, 75005 Paris, France; Laboratoire de Physique de l’Ecole Normale Supérieure (LPENS), CNRS UMR 8023, Ecole Normale Supérieure, Université PSL, Sorbonne Université, and Université Paris Cité, 24 rue Lhomond, 75005 Paris, France

**Keywords:** COVID-19, SARS-CoV-2, carbon dioxide, infection risk

## Abstract

Preventive measures to reduce infection are needed to combat the COVID-19 pandemic and prepare for a possible endemic phase. Current prophylactic vaccines are highly effective to prevent disease but lose their ability to reduce viral transmission as viral evolution leads to increasing immune escape. Long-term proactive public health policies must therefore complement vaccination with available nonpharmaceutical interventions aiming to reduce the viral transmission risk in public spaces. Here, we revisit the quantitative assessment of airborne transmission risk, considering asymptotic limits that considerably simplify its expression. We show that the aerosol transmission risk is the product of three factors: a biological factor that depends on the viral strain, a hydrodynamical factor defined as the ratio of concentration in viral particles between inhaled and exhaled air, and a face mask filtering factor. The short-range contribution to the risk, present both indoors and outdoors, is related to the turbulent dispersion of exhaled aerosols by air drafts and by convection (indoors), or by the wind (outdoors). We show experimentally that airborne droplets and CO_2_ molecules present the same dispersion. As a consequence, the dilution factor, and therefore the risk, can be measured quantitatively using the CO_2_ concentration, regardless of the room volume, the flow rate of fresh air, and the occupancy. We show that the dispersion cone leads to a concentration in viral particles, and therefore a short-range transmission risk, inversely proportional to the squared distance to an infected person and to the flow velocity. The aerosolization criterion derived as an intermediate result, which compares the Stokes relaxation time to the Lagrangian time-scale, may find application for a broad class of aerosol-borne pathogens and pollutants.

Significance Statement Making rational public health policy decisions to prevent the dominant routes of viral transmission requires a simple but quantitative assessment of the airborne transmission risk in public places, both indoors and outdoors. Here, we show that CO_2_ and aerosol viral particles disperse following the same law, which allows us to relate this aerosol transmission risk to the CO_2_ concentration. The results provide quantitative guidance useful to complement vaccination, treatment for the vulnerable patient population, and the test–trace–isolate strategy by a national ventilation plan, and gradual mandates of FFP2 respirators in indoor places, when the epidemic circulates above a threshold.

## Introduction

The SARS-CoV-2 pandemic enters its third year despite the design of highly effective vaccines, which induce circulating antibodies and systemic T- and B-cell responses that block viral spread and disease. Besides the lack of vaccination at the global scale, they do not establish immunity at mucosal surfaces against infection by variants such as Omicron (B.1.1.529), which present both increased intrinsic transmissibility and viral immune evasion. Widespread transmission therefore contributes to a degree of unpredictability in the evolution of the pandemic. As a consequence, vaccination must be complemented in the long term by effective public health policies contributing to suppress transmission at a low economic and social cost. To help making rational public health policy decisions, we propose here a method to measure the risk for long- and short-range airborne transmission of SARS-CoV-2, both indoors and outdoors.

Respiratory viruses and bacteria can be transported by droplets emitted by coughing or sneezing, which may cause symptomatic transmission, and during expiratory human activities such as breathing, speaking, or laughing, which may cause asymptomatic and presymptomatic transmission. Pathogens responsible for illnesses such as influenza, tuberculosis, measles, or SARS, initially carried by these droplets, can form an aerosol phase (1, 2) and cause airborne transmission by inhalation. The silent spread of SARS-CoV-2 by asymptomatic infected individuals, who do not cough nor sneeze, has been hypothesized as early as January 2020. Since June 2020, there is ample evidence that this virus is primarily transmitted through aerosols (3–6). This is particularly obvious in public places where face mask wearing has been mandatory (7) as the heavier, millimeter-sized droplets have a ballistic trajectory that is relatively insensitive to the presence of air and are stopped by all types of masks.

Many misconceptions regarding aerosols can be found in the medical and scientific literature. Clinicians often use an incorrect definition, still reported by the World Health Organization: aerosols would be particles smaller than 5 μm that settle slowly enough to be transported over a few meters (6, 8). As a consequence, problematic criteria are introduced, such as the distance after which a particle launched with an initial velocity in a fluid at rest stops (9), or the settling time of a single particle dropped from head height in still air (10). An aerosol is a locally homogeneous phase constituted of solid or liquid particles suspended in a gas, either by thermal fluctuations (for very small particles) or by turbulent fluctuations. The aerosol phase tends to homogenize and to diffuse over the whole available space by turbulent dispersion. The “slow settling” misconception therefore omits the fundamental constitutive mechanism of aerosols of any particle size: turbulence.

The infection risk has been modeled in a series of papers, starting from the seminal works of Wells and Riley (11, 12), which parametrize the infection risk as a function of the global air dilution and the disease infectiousness. The authors work upon the well-mixed hypothesis, in which air inside a room is instantly mixed by turbulence so that all people inside breathe the same air. Beyond this hypothesis, fluid dynamics models of indoor air circulation (13–17) and heterogeneous airflows produced by respiratory activity have been introduced (18–20). The Wells–Riley model has been extended to unsteady conditions by Rudnick and Milton (21), who have pointed out that CO_2_ could be used as a risk proxy (22). Regarding SARS-CoV-2, most models extend the Wells–Riley equation to account for unsteady conditions in ventilation or occupancy (23, 24) and to different viral emission rates depending on the respiratory activity (25), face coverings, and particle removal and inactivation (26–28). All of these models use well-characterized “super-spreading” events to derive estimates of the viral emission rate, or closed microsocieties such as cruise ships (29, 30).

These models have left three problems open up to now. (i) Can an analytical closed formula be derived, simple enough to be used to produce regulatory ventilation standards? (ii) Is the dispersion of CO_2_ and airborne viral particles governed by the same law? (iii) What is the law governing the short-range contribution to the transmission, both indoor sand outdoor?s Here, we propose a definition of the environmental risk of viral transmission in public spaces such as schools, offices, university lecture halls, museums, or shopping centers, but also outdoors. We report experimental and theoretical results showing that exhaled CO_2_ and airborne viral particles diffuse at the same rate. We finally derive a quantitative analytical model that relates this risk to the CO_2_ concentration.

## Airborne transmission mechanism

The main entry of SARS-CoV-2 virus is through the upper respiratory epithelium. To colonize a cell, an inhaled viral particle interacts through the spike protein—which is cleaved by a host cell protease, mostly the TMPRSS2 protease for the wild Wuhan-1 strain—with a host cell membrane protein, the ACE2 receptor. Cleavage of the spike protein is necessary for a conformational change so that it can effectively interact with the ACE2 receptor. This interaction leads to the formation of a virus–ACE2 complex that triggers the internalization of the virus inside the cell. It replicates its RNA molecule and produces the proteins required for self-assembly of new viral particles, which are released, leading to the colonization of neighboring cells (33).

An organism may become infected if a sufficient amount of viral particles interact with cells expressing both the TMPRSS2 protease and the ACE2 receptor and if the virus is able to hack into cellular mechanisms to produce and disseminate new virions. From the upper respiratory epithelium, which is the first tissue to be infected (nasal epithelium for viral strains before Omicron, but also throat epithelium for Omicron, due to a weaker dependency to the TMPRSS2 protease), the virus, embedded in mucus, is carried to the trachea, to the lungs or the esophagus, and finally to deeper organs. It has also the possibility to reach the brain and to colonize certain cells of the cortex.

When the virus is concentrated in the nasal cavity, it is disseminated via a mist of fine droplets of mucus or saliva dispersed by breathing, talking or singing. A sneeze or cough produces larger droplets containing viral particles (Figure S3). The evaporation of mucosalivary liquid droplets in the air is controlled by the ambient relative humidity RH and by its content in surfactants, proteins, and electrolytes. Initially, transport of water molecules from the droplets to the surrounding air is diffusive so that the drop squared radius decreases linearly in time. After a very short time, the droplet stabilizes at an equilibrium radius at which the viral particle is surrounded by proteins and water (10, 34–37). The influence of droplet chemical composition on the equilibrium radius is still poorly understood (38–40). The radius shrinks by a factor two to five (see Supplementary Material): droplets emitted during breathing, below 20 μm, are therefore submicronic or micronic and stay suspended in the air.

The transmission risk increases with the intake viral dose *d*, defined as the amount of infectious viral particles inhaled by a person, cumulated over time. *d* increases with the time of exposure to the virus, with the inhalation rate *q_e_*, i.e. the product of the breathing rate by the tidal volume (for light exercise, *q_e_* ≃ 0.5 m^3^/h), and with the concentration *C_i_* of infectious viral particles in the inhaled air. As a dose is a quantity of virus, it can be measured using quantitative RT-PCR and is then expressed in genome units (GU). However, it is better adapted to measure a dose by infecting a culture cell monolayer, in plaque-forming units (PFU). PEU measure the ability of viral particles to replicate and to be secreted by the chosen cell type, while qRT-PCR measures the number of RNA molecules and is not sensitive to replication potential. On generic Vero cells, the amount of virus needed on average to form one cell lysis has decreased from 1400 GU/PFU for the wild strain to 240 GU/PFU for the Delta variant (41).

The inhaled dose *d* is the product of two factors:

- a purely biological factor reflecting the exhalation flux of viral particles and the ability of the virus to infect a person;
- a purely physical factor ϵ reflecting the dilution of viral particles between exhalation and inhalation.

We will investigate these two factors independently in the next sections.

## Dispersion and transport of CO_2_ and viral particles

### Average concentration

**Fig. 1.**
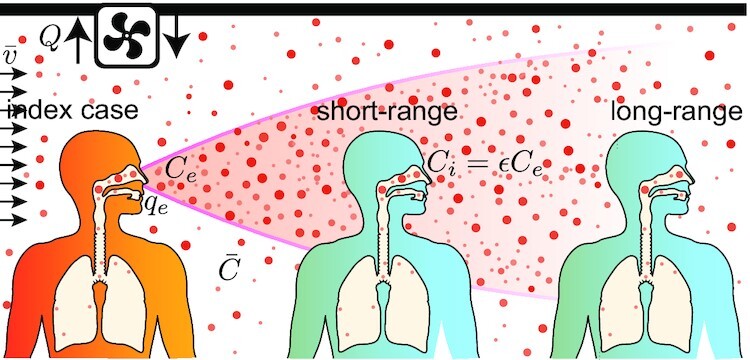
Viral particles are exhaled by an infected person at a concentration *C*_e_. They are dispersed by drafts (velocity $\bar{v}$), which lead to a decrease of their concentration. The dispersion cone must not be confused with the conical shape of high-speed jets in a fluid at rest (19, 31, 32). In an indoor space, viral particles are stored, which translates into a background homogeneous concentration $\bar{C}$, controlled by the ventilation rate *Q*.

In a public space where many people are gathered, let us single out two people: one is infectious and exhales viral particles and the second one inhales the air. The air exhaled by the infectious person is concentrated in viral particles, which are gradually diluted in the ambient air, in a way similar to the smoke of a cigarette. Outdoors, far from an infected person, the viral particle concentration vanishes. However, indoors, viral particles accumulate: the concentration in the wake of an infected person decays from its exhalation concentration *C_e_*, which is high, to a constant concentration $\bar{C}$ far away. The dilution factor ϵ is defined as the ratio of the viral concentration *C_i_* in the inhaled air and the viral concentration in exhaled air *C_e_* (Fig. 1). First, consider a closed room of volume *V* where there is a single person exhaling viral particles at a concentration *C_e_*. A ventilation of flow rate *Q* replaces exhaust air at the average concentration $\bar{C}$ by fresh air. The average concentration $\bar{C}$ obeys the conservation equation: $V \mathrm{d}\bar{C}/{\mathrm{d}t}= q_e C_e- Q \bar{C}$. It is a linear relaxation equation whose exponential relaxation time is *V*/*Q*. The average dilution factor tends towards the steady-state solution $\bar{\epsilon }= \bar{C}/C_e=q_e/Q$ (21).

### Dispersion

**Fig. 2.**
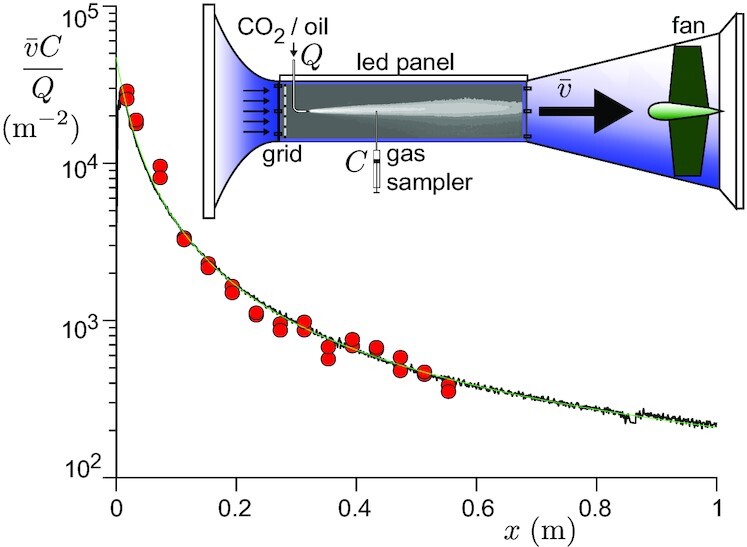
Rescaled concentration profile of 10-μm-size oil droplets (black solid line) and CO_2_ (red circles) in a wind tunnel experiment. The CO_2_ concentration along the axis is measured after sampling the gaz with a syringe. The oil droplet concentration is measured using the optical absorption of light. CO_2_ and oil aerosol are introduced successively at a rate *Q*, 20 cm downstream of the turbulence generating grid. The working velocity is $\bar{v}=9\,\,{\rm m/s}$. The green line is the best fit by Eqs. (1) and (2).

We have investigated experimentally and theoretically the turbulent dispersion of viral particles and CO_2_ in a turbulent flow of mean velocity $\bar{v}$, characterized by a root mean square velocity σ_*V*_. This corresponds to a generic situation where horizontal drafts (indoors) or wind (outdoors) dominate over thermal plumes, so that natural convection can be neglected. We have, for instance, shown in a recent paper that this was the case in the corridors of commercial malls (42). The dispersion of CO_2_ and of oil droplets of typical size 10 μm is studied separately, but in the same conditions, in the wind tunnel schematized in Fig. 2B. The smoke concentration field is measured using high-resolution pictures (7380 × 4920) with a 2 s exposure time (Fig. S1). The CO_2_ concentration profile $C^{{\rm CO}_2}(x)$ along the axis is measured after careful sampling of air using a syringe: the air is transferred into a chamber where a vacuum has been set, equipped with a CO_2_ nondispersive infrared sensor (Fig. S2).

As dispersion is slow compared to the axial convection, the axial coordinate *x* is equivalent to time $t=x/\bar{v}$. Transverse diffusion is controlled by random motion and leads, according to the central limit theorem, to a quasi-Gaussian radial profile of concentration (42). As the flux across a transverse section is equal to the source emission rate *q_e_C_e_*, the concentration field takes the form

(1) $$\begin{eqnarray*}
C=C_e\,\,\frac{q_e }{\pi \sigma _R^2 \bar{v}}\,\,\exp \left(-\frac{r^2}{2\sigma _R^2}\right)+\bar{C}.
\end{eqnarray*}
$$

The measurement of the dispersion radius σ_*R*_ as a function of *x* gives the law of spatial decay of the concentration.

Because of turbulence, the velocity of an elementary volume of fluid is correlated along its Lagrangian trajectory. This drives transported particle velocities to be correlated too. We model the dispersion as a Langevin process characterized by an exponentially decaying Lagrangian correlation function: $\overline{\mathbf {v^{\prime }}(t)\mathbf {v^{\prime }}(t+\tau )}=\sigma _V^2\exp (-\tau /{\mathcal {T}})$, where ${\mathcal {T}}$ is the Lagrangian integral time-scale. The dispersion of fluid particles injected at a source point at time *t* = 0 is given by Taylor’s theorem, which gives at time $t=x/\overline{v}$ (42)

(2) $$\begin{eqnarray*}
\sigma _R^{2}= \frac{2}{3}\sigma _V^2 {\mathcal {T}}^2 \left[\exp \left(-\frac{x}{{\overline{v}}{\mathcal {T}}}\right) +\frac{x}{{\overline{v}}{\mathcal {T}}}-1\right].
\end{eqnarray*}
$$

At large distances $x \gg \overline{v} \mathcal {T}$, it predicts a diffusive regime $\sigma _R \sim \sigma _V \left( {\mathcal {T}} x/ {\overline{v}}\right)^{1/2}$, the concentration along the axis (*r* = 0) decaying as 1/*x*. The most important dispersion takes place at short distances $x \ll \overline{v} \mathcal {T}$. Transport obeys a ballistic-like regime of the form $\sigma _R\sim \sigma _V x/ \bar{v}$, which corresponds to a fast decay of the concentration along the axis (*r* = 0) as

(3) $$\begin{eqnarray*}
C-\bar{C}=C_e\,\,\frac{q_e \bar{v}}{\pi (\sigma _V x)^2}.
\end{eqnarray*}
$$

### Face masks and respirators

In public places where face masks are mandatory, the aerodynamical contribution to ϵ must be multiplied by the product of the exhalation and inhalation mask filtration factors. All types of masks totally filter droplets of a fraction of millimeter. However, they have very different efficiencies between 0.1 and 0.5 μm, which is the range of equilibrium sizes of the smallest mucus droplets after evaporation. Mask efficiency presents a minimum around 0.3 μm, where the particles are large enough to make Brownian collection inefficient, and tiny enough to have small inertia, insufficient to make them leave the streamlines wrapping around the fibers (43). Filtration factors are determined by the material properties and the respiratory activity at play, but most importantly by proper mask fit. Cloth and surgical masks tend to be much looser fitted than respirators, thereby greatly reducing their filtration factor compared to what their fabric could achieve alone (44). Cloth masks have an effective filtration factor around λ = 0.70, on average (45, 46). Assuming they are well-fitted, surgical masks have an effective filtration factor λ = 0.28 (47, 48) and N95/FFP2 respirators λ = 0.10 (49, 50).

## Modeling the transmission risk

### Intake viral dose

Transmission risk assessment requires the estimate of the probability of infection under a given intake viral dose *d*. The simplest hypothesis is to assume an independent action of all inhaled replicable viral particles, which means that a single virus can initiate the infection. However, more than one is statistically needed, as the probability that a single infectious viral particle overwhelms the host immunity defences successfully is small, typically between 10^−3^ and 10^−2^ (41). For a person having inhaled an intake dose *d*, the probability law of infection *p*(*d*) takes the form

(4) $$\begin{eqnarray*}
p(d)=1-e^{-a d},
\end{eqnarray*}
$$

where the susceptibility *a* is the inverse of the infection dose defined, for each individual, as the intake dose for which the probability of infection is $1-1/e\simeq 63\,\,\%$. *a* is widely distributed across individuals, according to a probability distribution *f*(*a*). We denote by $\bar{a}=\int a f(a) \mathrm{d}a$ the population average of *a*. $\bar{a}^{-1}$ is the infectious dose, called the quantum of infection when used as a convenient unit for a quantity of infectious viral particles; the product $\bar{a} d$ is then the dose expressed in “quanta.” For the wild strain Wuhan-1, the infectious quantum has been estimated around 5.6 × 10^5^GU and 400 PFU (41). Importantly, the distribution *f*(*a*) includes the effect of vaccinal or infection induced immunization. The infectious quantum is indeed a population average over different immunity conditions.

### Relation between inhaled dose and viral exhalation rate

In the literature (15, 25, 26, 35, 51–53) devoted to airborne transmission of SARS-CoV-2, most authors have considered that the viral emission rate, i.e. the number of virus exhaled per unit time, is the product of the volume of mucus droplets emitted per unit time by the viral load in the nasal cavity for mucus droplets, in the throat for saliva droplets (28) or deeper in the lungs for respiratory aerosols. This excludes the possibility of a viral enrichment at the interface between mucosalivary fluid and air, which could lead to a viral content of droplets, which is not proportional to their initial volume.

**Fig. 3.**
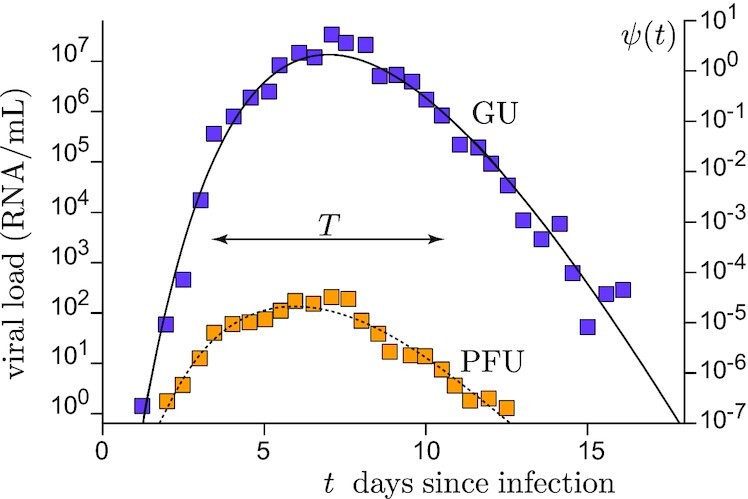
Viral kinetics of SARS-CoV-2. Average viral load in the nasal cavity as a function of time since infection from Killingley, *et al*. (59), using a strain close to Wuhan-1. Blue squares are obtained by quantitative RT-PCR and expressed in GU. Orange squares are obtained by counting lysis plaques and expressed PFU. Right axis: model dimensionless viral load ψ(*t*) as a function of time *t*, in days, after infection.

Infectivity is proportional to the concentration of infectious viral particles in the exhaled air, noted *C_e_*. It is assumed here to follow the same kinetics as the infectious viral load. It also depends on biological factors, which differ from one viral strain to the other. The viral kinetics results from the competition between viral replication and immune response. For simplicity, *C_e_* can be assumed to present the same temporal profile amongst patients:

(5) $$\begin{eqnarray*}
C_e=C_m\,\,\psi \left(t-t_c\right),
\end{eqnarray*}
$$

where *C_m_* is a characteristic concentration and *t_c_* the infection time. Both *C_e_* and *C_m_* are expressed in PFU/m^3^. The rescaled viral load curve ψ(*t*) is dimensionless and can be considered as the average over the subpopulation considered. Typically, ψ increases exponentially in the presymptomatic period and decreases exponentially at long times, as shown in Fig. 3 (54–56). We choose here the following normalization for ψ(*t*), which provides an unambiguous definition of the mean contagious time *T*

(6) \begin{eqnarray*} \int _0^\infty \psi (t)\mathrm{d}t=T, \quad \int _0^\infty t\,\,\psi (t)\mathrm{d}t=T^2. \end{eqnarray*}

The characteristic viral concentration *C_m_* is highly variable among infected people. Moreover, it depends on the vaccination status, on former infections, on the quality of the immune system, etc. In order to consider an average over all statistical realizations, we introduce the probability density function *g*(*C_m_*), which is typically log-normal. Depending on the hypotheses used to extract *C_m_* from sparse RT-qPCR measurements, its dispersion is found between 0.1 to 1 log_10_ copies/mL above and below average (55, 57, 58).

The inhaled dose can be expressed as

(7) $$\begin{eqnarray*}
d= \int dt\,\,\lambda ^2\,\,q\,\,C_i=\int dt\,\,\lambda ^2\,\,\epsilon \,\, q \,\,C_m.
\end{eqnarray*}
$$

The key quantity is the mean integrated viral emission $\bar{h}$, which accounts for all the biological part of the risk. By definition, it is the dose in “quanta” exhaled by a patient during the entire infectious period:

(8) $$\begin{eqnarray*}
\bar{h}=\int dt \bar{q} \bar{a} C_e=\bar{q} \bar{a} \bar{C}_m T.
\end{eqnarray*}
$$

It is defined as an average over the subpopulation attending the public space considered and may depend on the particular activity taking place in the public space through the mean inhalation rate $\bar{q}$. The integrated viral emission $\bar{h}$ is between 450 and 500 quanta for Wuhan-1, 800 for Alpha, 1500 for Delta, 2,800 for Omicron BA.1, and 4,700 for Omicron BA.2 (41).

### Environmental risk

We define the transmission risk of a public space as the average number of infections *r* that an infected person staying in for an infinitely long time would cause, on average. This public space contributes to epidemic decay if *r* < 1 and to its growth otherwise. *r* characterizes the environmental conditions and behaviors in the public space. We consider a public place hosting *N* people during a given period of time, among which *M* are infectious. The epidemic incidence *I*(*t*) is the fraction of a given population infected per unit time. In practice, the fraction of the population *I*(*t*)δ*t* infected between *t* and δ*t* is measured with δ*t* = 1 day, although conceptually δ*t* is infinitesimal. The prevalence *P* is the fraction of this population currently infectious and is related to *I* by the equation

(9) $$\begin{eqnarray*}
P(t)=\int _{-\infty }^0 I(t^{\prime }) \psi (t-t^{\prime })\,\, \mathrm{d} t^{\prime }.
\end{eqnarray*}
$$

On average, the number *M* of infectious people among *N* is $\bar{M}=PN$. Under the Poissonian hypothesis, the mean number *rM* of people infected reads

(10) $$\begin{eqnarray*}
rM=\sum _{i=1}^{N-M}\left(1-e^{-a_{i} d_{i}}\right),
\end{eqnarray*}
$$

where *d_i_* is the intake dose of the individual labeled *i* while 1/*a_i_* is their infection dose. In the case where all *N* people are statistically subjected to the same intake dose *d_i_* = *d*, the risk *r* reads

(11) $$\begin{eqnarray*}
r=(N-M) \int _0^\infty f(a) \left(1-e^{-a d}\right)\,\, \mathrm{d} a.
\end{eqnarray*}
$$

The quantity ρ(*d*) = ∫*f*(*a*)(1 − *e*^−*ad*^) d*a* is the probability to get infected when an intake dose *d* is inhaled and is called the dose response function. We consider now the limit where the intake dose, expressed in quanta $\bar{a} d$, has a very low probability of being larger than 1. This excludes super-spreading events, which occur when one infectious person (or several) with a large exhaled concentration *C_m_* attends an under-ventilated place, leading to multiple simultaneous infections. Then, performing the linearization 1 − exp ( − *ad*) ≃ *ad*, the equation simplifies into $\bar{Z}=(N-M) \bar{a} d=M(N-M) \bar{a} \epsilon \bar{q} \bar{C}_m T$. The average number of secondary infections $r=\bar{Z}/M$ is therefore proportional to the biological factor $\bar{h}$, to the dilution ratio ϵ:

(12) $$\begin{eqnarray*}
\bar{r}=(N-M)\epsilon \bar{h}.
\end{eqnarray*}
$$

For the same concentration of viral particles in the air, the transmission risk increases with the number of people (*N* − *M*) susceptible to be infected as they all have an equal individual risk of being infected.

## Results

### Transport of respiratory aerosols

Does the dispersion of CO_2_ and virus laden droplets obey the same law? To answer this question, we compare experimentally and theoretically the turbulent dispersion of an aerosol of oil droplets and of CO_2_ in a controlled turbulent flow. Fig. 2A shows that the concentration decays of the gaz and of the aerosol superimpose without any adjustable parameter. This remarkable result is highly nontrivial. Inertial particles disperse at the same rate as massless particles if the particle velocity correlation is close enough to the fluid velocity correlation function. Inertia acts as a low-pass filter of the fluid velocity, with a cut-off time controlled by the Stokes times τ_*S*_ = ρ_*p*_*d*^2^/18η, which is the particle response time to a change in the fluid velocity (60).

The quantitative criterion for the aerosolization of particles of different sizes must compare the particle inertia to the dispersion by turbulent fluctuations. The stopping distance after which a droplet exiting the upper airways with a large initial velocity stops in a fluid at rest (43) includes inertia but ignores turbulent particle diffusion. Inertia acts all along the particle trajectory: the effect of particle size on turbulent dispersion is to make large enough particles slow-paced at changing their velocities, so that they leave the gas streamlines and decorrelate from the flow. We have shown here that the relevant turbulent property to which inertia must be compared is the Lagrangian time-scale $\mathcal {T}$. This experimental result is in agreement with correlations functions found from direct numerical simulations of particles in turbulence (61). Particles form an aerosol phase whenever turbulence can homogenize their concentration. Settling gradually depletes the phase, but does not determine whether droplets should be regarded as individual ballistic particles or as an Eulerian phase. Settling times incorrectly assume that the air is still. Our experiments show that gravity is not the relevant physical mechanism that separates small particles from large particles, but the balance of inertia and turbulence is, and the dimensionless ratio $\mathrm{St} \equiv \tau _S/\mathcal {T}$ is the correct way of defining the Stokes number for the dispersion problem (see Supplementary Material for a derivation). If the Stokes number St is much smaller than 1, particles presents a negligible inertia, and are therefore aerosolized and dispersed according to Eq. (2).

In the wind tunnel used in our experiments, we measured $\mathcal {T} = 9\,\,{\rm ms}$ (42). The cross-over above which inertia becomes important ($\tau _S = \mathcal {T}$) is therefore expected for a droplet diameter ∼50 μm much larger than the oil droplet diameter. For a meter scale ventilated room, the Lagrangian time-scale $\mathcal {T}$ is in the range of 10^2^ ms and the cross-over droplet diameter is larger than ∼100 μm. To conclude, CO_2_ and viral particles are dispersed at the same rate so that the dimensionless factor ϵ can indeed be determined quantitatively from the measurement of the CO_2_ concentration.

Fig. 2A shows that Eqs. (1) and (2), with a single relaxation time-scale $\mathcal {T}$, perfectly fit the data. This result is particularly subtle and surprising. At such high Reynold numbers (between 10^5^ and 10^6^), turbulence is fully developed in space. According to the energy cascade picture of turbulence, kinetic energy is injected at large scale and dissipated by viscosity at small scale. At intermediate length scales, called the inertial range, energy is transferred by inertial effects (62), which cause nontrivial velocity correlations between two points separated in space. The typical flow evolution time-scale at the dissipation scale is called the Kolmogorov time τ_*K*_ and is used in the turbulence community to define a different particle Stokes number St (61). Indeed, τ_*S*_/τ_*K*_ is relevant for the pair dispersion of particles (63, 64). The transport of a single particle is a Lagrangian problem (42), and therefore does not involve the Kolmogorov time, but rather the Lagrangian decorrelation time $\mathcal {T}$. Surprisingly, for the Reynolds numbers accessible numerically and experimentally, Lagrangian two-points correlation functions in time do not feature any inertial range, while Eulerian two-points space correlation functions do (61). Fig. 2 shows that we do not find any signature of an inertial range on the Lagrangian correlation function in time either. We do not observe any signature of a power-law regime, but instead, a simple ballistic-like regime. Simple arguments à la Kolmogorov would rather predict a much faster power-law decay of the velocity correlation function, leading quickly to a diffusive law and therefore of a decay of $C-\bar{C}$ as *x*^−1^.

Experiments with a volunteer breathing through the mouth (42) have shown that viral particles are injected at a length scale *a* ≈ 0.3 m, set by the head size, which is significantly smaller than the turbulent integral scale. Eq. (3) can directly be used for exhalations, by changing *x* to *x* + *a*, so that

(13) $$\begin{eqnarray*}
\epsilon =\bar{\epsilon }+\frac{q_e \bar{v}}{\pi \sigma _V^2 (x+a)^2}.
\end{eqnarray*}
$$

The aerosol concentration therefore present a fast decay as the inverse squared distance *x* to the infected person. It is inversely proportional to the wind speed, at constant fluctuation rate $\sigma _V/\bar{v} \sim 10^{-1}$. Eq. (13) constitutes a central result of this paper, as it controls the spatial structure of the transmission risk outdoors.

### Transmission risk and CO_2_ concentration

The *N* people present exhale CO_2_ while only the *M* infected people emit viral particles. As a consequence, the expression of ϵ in terms of the average CO_2_ concentration 〈*C*〉 also contains a factor *N*:

(14) $$\begin{eqnarray*}
\langle \epsilon \rangle =\frac{\langle C^{{\rm CO}_2}\rangle -C^{{\rm CO}_2}_\infty }{N\,\,C^{{\rm CO}_2}_e},
\end{eqnarray*}
$$

where *C_e_* ≃ 37,500 ppm is the average CO_2_ concentration in exhaled air. It must be emphasized that Eq. (14) does not assume any relationship between CO_2_ and virus emission rates and is equally valid for breathing, talking, or singing. It only hypothesizes that airborne viral particles are inhaled. The two factors balance each others in the transmission risk, which reads, after averaging over *M*: $\bar{r}=\lambda ^2 (1-P)N \langle \epsilon \rangle \bar{h}$. The prevalence is in general much smaller than 1 so that we obtain the final formula relating the viral transmission risk to the CO_2_ concentration:

(15) $$\begin{eqnarray*}
\boxed{\bar{r}= \lambda ^2 \frac{\left \langle C^{{\rm CO}_2} \right \rangle -C^{{\rm CO}_2}_\infty}{C^{{\rm CO}_2}_e}\bar{h}}
\end{eqnarray*}
$$

This equation is remarkable: the dependence of the environmental risk, which characterizes the creation of transmission chains, with respect to the volume of the room, the ventilation, and the occupancy number are all encoded in the space-averaged CO_2_ concentration 〈*C*〉. The result is more subtle than it seems at first sight. Indeed, the *N* occupants of a public space all exhale CO_2_ but only the *M* infected ones exhale virions. Let us compare the risk of a well-ventilated lecture hall (say, at 750 ppm of CO_2_), with 50 students, to the risk if the same students are spread out in two conventional rooms with the same CO_2_ concentration. Obviously, all other things being equal, the probability of a student being infectious is twice lower when 25 students are grouped together, rather than 50. However, since the ventilation must be proportional to the room occupancy to keep the CO_2_ level, the viral particles are twice more concentrated in the small room and therefore the inhaled dose doubles. Under the above assumption, the average number of people infected is the same, although the risk is distributed differently.

The average CO_2_ concentration $\langle C^{{\rm CO}_2} \rangle$ can be defined to take into account both the long-range dilution $\bar{\epsilon }$ and the short-range contribution, present both indoor and outdoor, due to the higher aerosol concentration in the wake of infectious people. To achieve this, the CO_2_ concentration must be averaged over the surface where one more person would stand in the public place. The reasoning is the same as before. Each time a CO_2_ molecule reaches the measurement point, there is a probability *M*/*N* that it has been exhaled by one of the *M* infectious people. In that case, viral particles follow the same path as CO_2_ molecules. The dilution between inhaled and exhaled viral particle concentrations is still valid, but only if averaged over the possible permutations of the *M* infectious people amongst the *N* people. Again, the transmission risk is distributed very differently in the wake of infectious and noninfected people but the average remains correct. The quantitative determination of the aerosol transmission risk is directly applicable to outdoor spaces, where it is limited to this dispersion cone in the wake of infected people. The short-range transmission risk outdoors is generally smaller than indoors, due to larger air flow velocities.

Let us illustrate on two examples the consequences of Eqs. (3) and (13). Consider a shopping mall corridor in which we have measured the air draft velocity to be typically 0.2 m/s. This corresponds to an extra CO_2_ concentration of 160 ppm at 1 m, of 50 ppm at 2 m, of 10 ppm at 5 m. Consider now an outdoor situation with a 1 m/s wind. This corresponds to an extra CO_2_ concentration of 30 ppm at 1 m, of 10 ppm at 2 m, and of 2 ppm at 5 m (Fig. S7).

**Fig. 4.**
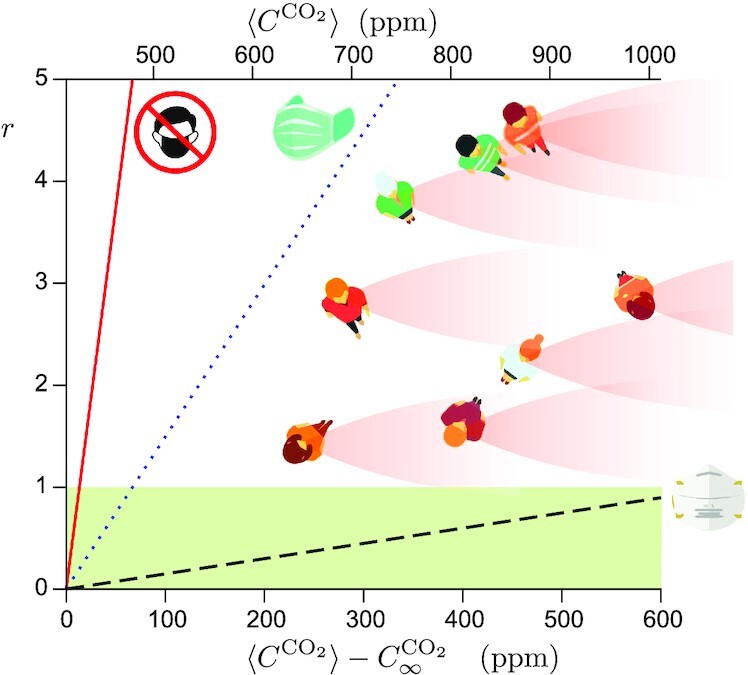
Airborne transmission risk *r* as a function of the concentration excess $\langle C^{\mathrm{CO}_2}\rangle - C^{\mathrm{CO}_2}_\infty$ for the Omicron BA.1 strain ($\bar{h} = 2800\,\,{\rm quanta}$). Solid line: no face mask. Dotted line: current level of mask-wearing in public, when mandatory (λ^2^ = 0.2, Fig. S4). Dashed line: FFP2 respirator (λ^2^ = 0.02). Inset: schematic showing the spatial distribution of short-range transmission risk in a public space. $\langle C^{\mathrm{CO}_2}\rangle$ is by definition the space average of the CO_2_ concentration over all places where a further person would spontaneously stand.

Fig. 4 shows the linear relationship between the aerosol transmission risk and the CO_2_ average concentration for different face masks and viral strains. The natural risk threshold is *r* < 1, below which an infected person infects on average less than one other person, making the epidemic recede. With the current level of mask-wearing in public (Figs. S4 and S5), which leads to a small filtration factor λ^2^ = 0.2, the maximum excess CO_2_ concentration should be 70 ppm. This corresponds to a CO_2_ concentration of at most *C* = 490 ppm. If the risk is large but ventilation cannot be changed, N95/FFP2 respirators could be mandated. Social acceptability of such mandates could be managed by targeting first high-risk spaces, such as public transportation (65), then gradually expanding the scope of nonpharmaceutical interventions based on reproduction number and prevalence thresholds, following transparent and planned rules. If respirators were mandated, the factor λ^2^ would be decreased by 50 and the maximum excess CO_2_ concentration should be 670 ppm. The risk becomes smaller than 1 for standard ventilation conditions. Without mandatory face masks, the maximum excess CO_2_ concentration should be *C* = 13 ppm. This is far too small to be achievable in practice. Ventilation with fresh air is energy consuming, both in winter and during heat waves. It is therefore important to complement ventilation with HEPA filters or UV-C neon lights air purifiers (Fig. S6), which are already used reliably and regularly (66), are cost-effective techniques for destroying nucleic acids, DNA or RNA from bacteria, viruses, or other microorganisms present in the air.

## Discussion

We have obtained, using controlled approximations, a very simple expression showing a linear relationship between airborne transmission risk and CO_2_ concentration. Several effects have been neglected, which we discuss here.

The effect of gravity is to transport droplets vertically at the settling velocity. It can be neglected when the latter is small in front of turbulent velocities. Typically, the 10 μm oil droplets settle at 3 mm/s. For the wind tunnel used here, the fluctuating velocity is typically σ_*V*_ ∼ 0.7 m/s, which corresponds to a cross-over droplet diameter, above which settling is dominant, of 150 μm. For a meter scale ventilated room, the cross-over diameter remains of order 10^2^ μm. For an aerosol of viral particles, deposition is therefore negligible, as can be checked from models taking it into account (23, 25, 26). As evaporation is very fast, the effect of gravity is at worst transient and limited to the largest droplets.

We assume that dispersion is controlled only by the ambient air flow, and not by expiratory air flows. Unmasked coughs and sneezes can create large velocities that dominate over ambient transport, in which aerosols are exhaled in a buoyant jet that gets mixed at its boundaries (18, 67). Face masks greatly reduce expiratory velocities, so that transport by the ambient flow is recovered 20 cm away from the source (68–70). Our work is particularly applicable outdoor, where there is no mixing but rather a horizontal turbulent flow, and wind velocities easily dominate over exhalation (Fig. S7). Measurements of crowds taking into account head orientation, crowd density and walking velocity show that horizontal velocities completely control the transmission risk (71, 72).

Virions transported in an aerosol phase are gradually degraded by the damage done by antiviral proteins in mucosalivary fluid (73) and by UV-B radiation in sunlight or by UV-C. The inactivation rate of enveloped, airborne viruses such as SARS-CoV-2 depends on the physicochemical composition of the aerosol droplets, relative humidity RH, and temperature in ways that are still unclear. Relative humidity plays an important role: the inactivation rate has a maximum at high RH, reaches a minimum at intermediate RH then increases again for lower RH (38, 74–76). This suggests that virions can associate with proteins which protects them both from dessication and antivirals (39). Ignoring viral inactivation is our main simplifying assumption outdoors, as sunlight may make it much faster. Inactivation can be modeled as a relaxation process of time-scale τ, once *C* is defined as the concentration of replicable viral particles: $\mathrm{d}C/{\mathrm{d}t}= (q_e C_e- Q \bar{C})/V - \bar{C}/\tau$. The dilution factor ϵ and the transmission risk are therefore divided by 1 + *V*/(*Q*τ). The assumption made before corresponds to the regime where *Q* ≫ *V*/τ. As τ is on the order of an hour, this condition requires at least several “air changes per hour” (ACH) to be satisfied, which is the case in correctly ventilated public spaces. In steady state, measuring ϵ is therefore equivalent to measure both occupancy and ventilation rate. In transient conditions, often found in large public spaces, this approximation fails.

The mean integrated viral emission $\bar{h}$ has three sources of variability. The most important one is the variation of this biological factor between different public spaces, depending on the type of respiratory activity taking place there and on the characteristics of the population attending it. For breathing, the respiratory rate *q_e_* can vary by a factor 2 to 3, depending on activity level and physiological characteristics. Here, it should be understood as the average value for the level of activity inside the public space, e.g. its typical value at rest for a theater, light exercise for a shopping mall where people walk, and exercise for a sport facility.

The viral exhalation rate dependence on respiratory activity, talking, and singing is scarcely known. It has been estimated that talking increases it by a factor ∼10 (77, 78). However, few speech samples were culture-positive, either due to protocol limitations or because the virus may have lost some of its ability to replicate when exiting through the mouth (77, 79). It is therefore difficult to define $\bar{h}$ for each public space, and it must be averaged over the whole society. Individual exhalation rates, peak viral load, and individual infection doses are also broadly distributed, which creates another source of variability. There is also a variability originating from the number of contacts between individuals. Individuals who have many contacts get infected earlier and are thus removed from the pool of susceptible individuals, which decreases $\bar{h}$ over time.

The last simplifying assumption is a small intake dose $\bar{a} d$ (in quanta), which corresponds to a small dilution factor ϵ. Eq. (14) shows that this hypothesis corresponds to a situation, which is either well ventilated or with a large number of people. This is generally the case for public spaces. By contrast, domestic spaces in winter generically gather few people in bad ventilation conditions, during long periods of time. This assumption excludes super-spreading events in which almost everyone gets infected because of these large deviations and poor ventilation.

## Conclusion

In this article, we have introduced an effective definition of the airborne transmission risk *r* associated with a public space, defined as the average secondary infections per initially infected person. Under the commonly accepted hypothesis of no cooperation between virions, the risk is computed in the limit where it is low. It is related to the integrated quantum emission $\bar{h}$, to the mask filtration factor λ^2^, and to the dilution factor ϵ between exhaled and inhaled air. $\bar{h}$ accounts for all biological aspects and depends on the viral strain considered but also on the characteristics of the subpopulation attending the public space considered. In that sense, $\bar{h}$ takes into account the average respiratory activity in the public space in question. ϵ accounts for all hydrodynamical aspects and is decomposed into an average contribution controlled by ventilation and a spatially dependent contribution, localized in the dispersion cone of infected people, controlled by turbulent air flows. Indoor and outdoor spaces both present a risk of airborne transmission at short range, in the dilution cone of the exhaled breath. However, indoor, enclosed spaces only present a risk of airborne transmission at long range. Eq. (13) provides the answer to our initial question (iii) as it provides the law governing the short-range contribution to the transmission, both indoors and outdoors.

Eq. (15) constitutes a central result of the article, as it provides an answer to our initial question (i). Indeed, it incorporates the ventilation flow rate, the room volume, and the occupancy number into a single measurable quantity: the CO_2_ concentration. The disappearance of the occupancy *N* from Eq. (15) at large *N* is nontrivial and comes from two factors balancing each other: on the one hand, CO_2_ is exhaled by all individuals present, and not only by people infected by the virus; on the other hand, the transmission risk increases linearly with the number of people susceptible to be infected. As the risk is proportional to the CO_2_ concentration, the acceptable CO_2_ concentration below which *r* < 1 can be readily adjusted as new variants with higher $\bar{h}$ keep on appearing, based on a feedback using the measured reproduction number.

The simplicity of Eq. (15) is based on the third important result derived here, both experimentally and theoretically: the dispersion of CO_2_ and airborne viral particles is governed by the same law, for usual Reynolds numbers (question ii). The quantitative criterion is a small enough Stokes number $\mathrm{St} \equiv \tau _S/\mathcal {T}$ defined as the ratio of the Stokes time to the Lagrangian integral time. This result, albeit simple in appearance, results from a subtle effect of Lagrangian turbulence. This has important implications in determining infectivity times: as settling is not the relevant physical mechanism for aerosol transport, the infectivity of particles between 20 and 100 μm could be underestimated by models in which the air is still (10). We are not aware of previous simultaneous measurements of gas and particles in the same controlled flow.

The transmission of SARS-CoV-2 in public spaces is predominantly airborne. In complement to vaccination, treatments for the vulnerable patient population and the test–trace–isolate strategy, the infection risk can be reduced by a combination of four collective actions:

- ventilation with a sufficient fresh air flow rate per person to reduce the long-range risk;
- monitoring CO_2_ to measure the risk and adjust practices;
- air purification to complement ventilation where needed;
- turbulent dispersion, distancing, and reduction of static crowds to reduce the short-range risk, both indoors and outdoors.

Ventilation guidelines or regulations in public spaces are centered around balancing thermal comfort with energy consumption and perceived air quality, not infection prevention (80), in part because the physical basis of airborne transmission was poorly understood (6, 8). The approach presented here defines an unambiguous transmission risk, encoding most hydrodynamical aspects into a single measurable quantity. It is applicable to all airborne pathogens and to many flow configurations. It also defines a maximum acceptable risk: *r* = 1 is the threshold above which the epidemic continues to propagate. In that sense, it is a step toward ventilation regulation for infection prevention.

The transmission risk model outdoors is extremely easy to implement in practice. Combining Eqs. (12) and (13), we find

(16) $$\begin{eqnarray*}
r=\frac{\bar{h} q_e \bar{v}}{\pi \sigma _V^2 (x+a)^2}.
\end{eqnarray*}
$$

At the request of municipal services, we have applied it to the “Canal Saint-Martin” in Paris, France, the popular banks of a canal along which many people eat and drink. Measuring the mean distance between visitors (typically 1 m), we find that when the wind is aligned with the banks, *r* is below 1 for the Wuhan-1 strain and for wind speeds above 1.5 m/s. However, the wind is not always perfectly aligned, and fluctuations in the wind direction lead to dispersion over the canal, and therefore reduced risk. Recommendation to local policymakers was to emphasize the lower risk outdoors but simultaneously to mandate face masks during the very few days where there is not enough wind to disperse viral particles. Using a slightly revised dispersion formula, adapted to a cycling group, we have similarly been able to establish that the transmission risk is low in the Tour de France peloton, but not indoors, in the hotels hosting the cyclists.

We propose that the paradigm of airborne infection prevention policies could shift from a set of independent nonpharmaceutical interventions (mask mandates, occupancy limits, social distancing) to a standard of risk, translated to a maximum acceptable CO_2_ concentration. Each public place has several options to meet this imposed standard, itself depending on the local community transmission, by lowering the maximum occupancy or by investing in better ventilation, by mandating masks or respirators, or by purifying the air with HEPA filters or UV-C flash lights (Fig. S6). Restrictions can be gradually added at different reproduction rates and prevalence thresholds by targeting high-risk spaces first to balance social acceptability. Education on good quality masks and their wider availability could greatly reduce risk in public transportation (65) and shopping malls. If, after targeting first high-risk spaces, the global reproduction rate is still above 1, restrictions can be expanded to lower-risk spaces, until the epidemic recedes. It would also be possible, for example, to mandate that mechanical ventilation in public buildings have two flow rates: one that allows for ordinary air renewal, keeping the CO_2_ concentration below, say, 1,000 ppm, and another much more powerful one designed for a drastic lowering of pathogen concentrations in the air, despite energy or comfort costs. Based on the results presented here, French shopping malls have successfully used smoke extractor fans, renewing the air in 5 min (12 ACH), to that effect since May 2021. Insofar as smoke extractors are compulsory in this type of public spaces, this has not resulted in any additional costs.

## Supplementary Material

pgac223_Supplemental_Files

## Data Availability

The data supporting the findings of this study are available within the article and its supplementary material.
