# Supplementary material for: Risk assessment for long- and short-range airborne transmission of SARS-CoV-2, indoors and outdoors
Source: PNAS Nexus. 2022 Oct 6;1(5):pgac223. doi: 10.1093/pnasnexus/pgac223 (PMC9802175; doi:10.1093/pnasnexus/pgac223)
Supplement: pgac223_Supplemental_Files [file pgac223_supplemental_files.zip › PNASNEXUS-PNASNEXUS-2022-00550-s03.pdf]

PNAS Nexus, 2021, pp. 1–9

doi: DOI HERE
Advance Access Publication Date: Day Month Year
Paper

PAPER

Risk assessment for long and short range airborne transmission of SARS-CoV-2, indoors and outdoors (Supplementary Information)

Florian Poydenot,[a] Ismael Abdourahamane,[a] Elsa Caplain,[a] Samuel Der,[a] Jacques Haiech,[b] Antoine Jallon,[a] Inés Khoutami,[a] Amir Loucif,[a] Emil Marinov[a] and Bruno Andreotti[a,*]

[a]Laboratoire de Physique de l'Ecole Normale Supérieure (LPENS), CNRS UMR 8023, Ecole Normale Supérieure, Université PSL, Sorbonne Université, and Université de Paris, 24 rue Lhomond, 75005 Paris, France and [b]Cogitamus Laboratory and CNRS UMR 7242 BSC, 300 Bd Sébastien Brant, CS 10413, 67412 Illkirch Cedex

*To whom correspondence should be addressed: andreotti@phys.ens.fr

FOR PUBLISHER ONLY Received on Date Month Year; accepted on Date Month Year

Abstract

This Supplementary Information contains an expanded version of the methods.

Key words: COVID-19, SARS-CoV-2, carbon dioxide, infection risk

Experimental methods

Wind tunnel

Dispersion of aerosols and CO_2 is studied in a suction-flow wind tunnel with a 120 × 23 × 23 cm test section. A turbulence generating grid (3×3 grid of 7×7 cm squares separated by 1 cm wide bars) is place after the air inlet contraction section, 30 cm upstream of the test section. Wind velocity is measured with a hot-wire anemometer (test 405i). The flow Reynolds number is between 10^5 and 10^6.

Aerosol measurements

Aerosols are produced from heated oil vapor injected at a controlled rate and power through a 6 mm nozzle. A cloud of droplets nucleates at a few millimeters downstream. The test section is illuminated from above by a LED array with a diffuser. The cloud is made dilute enough so that double scattering is negligible and the light intensity scattered is linear with respect to the local aerosol concentration. High resolution pictures with a 1 or 2 s exposure time are taken with a Digital Single-Lens Reflex camera. In order to keep the image data linear with respect to the light intensity, the raw images files are debayered using a bilinear approximation and no further image processing is performed. We keep only the green channel to measure the light intensity. We average sets of 10 pictures taken in the same conditions to achieve a satisfying statistical convergence. Residual background light is removed by substracting the image of the tunnel without droplets. The illumination intensity profile along the tunnel axis $I_0(x)$ is calibrated using a diffusive object. $I_0(x)$ is flat, except at the start and the end of the measurement section where it decreases because of greater distance to the illumination source.

The intensity $\mathcal{I}(x, y)$ of a single pixel is an integral over the z axis of the scattered intensity, which is Gaussian as the concentration is Gaussian along a transverse section. Integrating it over z, the image intensity field $\mathcal{I}(x, y)$ is Gaussian along y:

$$\mathcal{I}(x, y) = I_0(x)\mathcal{C}(x)\sigma_R(x) \exp\left(-\frac{(y - y_0(x))^2}{2\sigma_R^2(x)}\right) \quad (1)$$

As shown on Figure S1, we fit each transverse pixel line over y by this corrected Gaussian profile to extract $\mathcal{C}(x)$, which is proportional to the concentration on the axis, and the radius $\sigma_R(x)$. $\mathcal{C}(x)\sigma_R^2(x)$ is a constant independent of x: conservation of mass is respected by our measurement technique.

© The Author 2021. Published by Oxford University Press. All rights reserved. For permissions, please e-mail: journals.permissions@oup.com

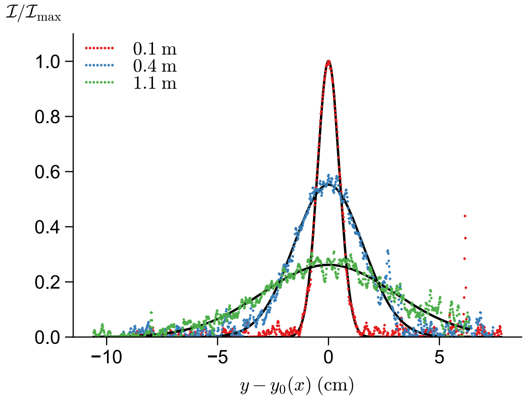

Fig. S1. Transverse intensity profiles at various distances from the oil outlet. The best fit by a Gaussian gives $\mathcal{C}(x)$, $\sigma_R(x)$ and $y_0(x)$.

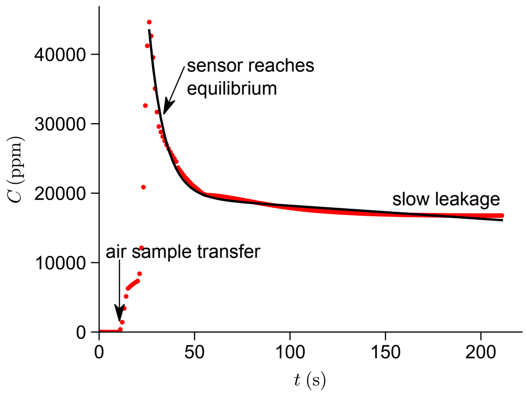

Fig. S2. Red dots: typical measured CO_2 concentration inside the vacuum chamber. The concentration rises rapidly as CO_2 is introduced and passes by the sensor, then the sensor equilibrates with an exponential decay. There remains a very slow decay, linear in time, due to leakage and adsorption. Solid black line: best fit by an exponential decay superimposed with a linear decrease in time.

CO_2 measurements

Solid CO_2 pellets are sublimated inside a pressurized container heated with a controlled power. A variable area flow meter controls the gas injection rate. The gas is injected at room temperature into the test section. CO_2 is then sampled along the dispersion cone axis by slowly suctioning air into a 60 mL syringe with a long needle. Three replicates are taken at the same point. A laser beam pointing along the flow axis helps position precisely the needle tip. After sampling, syringes are sealed until analysis.

The content of the syringe is transferred into a 300 mL chamber equipped with a non-dispersive CO_2 infrared sensor with a range of 0–50 000 ppm and with an absolute pressure sensor. Using a vacuum pump, a primary vacuum is made in the sealed chamber. Then, the 60 mL sample is sucked into the chamber. Finally, nitrogen is immediately added to the chamber to reach the ambient pressure. The CO_2 concentration is recorded as a function of time. After a few minutes, the sensor equilibrates and the concentration reaches a steady state. Figure S2 shows a typical temporal evolution of the measured CO_2 concentration. It rises rapidly as the sensor is near the entrance of the chamber, then the concentration homogenizes inside and the sensor relaxes to equilibrium with an exponential decay. There remains a small amount of leakage and adsorption that we fit with a linear decrease in time.

Since we measure its volumetric injection rate Q, the absolute volumic concentration of CO_2 is therefore known along the axis. This is not the case for the oil droplet aerosol, where only the relative concentration is known. The measured concentration on the axis $C_M(x)$ can be related to the transverse concentration profile:

$$C(r,x) = C_M(x)e^{-r^2/2\sigma_R^2} \tag{2}$$

Conservation of mass imposes that

$$\int_0^\infty 2\pi r \mathrm{d}r C_M e^{-r^2/2\sigma_R^2} = 2\pi\sigma_R^2(x)C_M(x) \tag{3}$$

is set by the injection rate, i.e.

$$2\pi\sigma_R^2(x)C_M(x) = \frac{Q}{\overline{v}} \tag{4}$$

We therefore plot $\overline{v}C_M/Q$ for CO_2 and $1/2\pi\sigma_R^2$ for oil droplets.

Turbulent dispersion: Lagrangian approach

Turbulent diffusion, like many diffusive processes (1), can be seen through two complementary lenses: a Langevin equation describing particle dynamics and a Fokker-Planck equation describing the spacial evolution of the concentration field. We develop these two approaches here. As turbulence acts as a fluctuating noise, in both cases we use the Reynolds decomposition (2) to split any field or dynamical variable A into its ensemble average over many realizations of the flow $\overline{A}$ and its fluctuating part with zero mean A': $A = \overline{A} + A'$.

We first consider a single particle of diameter d, density ρ_p, in a fluid of density ρ_f and viscosity η. Turbulent diffusion for particles without inertia is a correlated Brownian motion (3–5). The simplest form of a Langevin equation that leads to a finite correlation time gives an exponentially decaying velocity correlation function (1, 6–8):

$$\overline{\mathbf{v}'(t)\mathbf{v}'(t+\tau)} = \sigma_V^2 \exp(-\tau/\mathcal{T}) \tag{5}$$

The correlation time $\mathcal{T}$ is called the Lagrangian integral time scale, defined in the general case as:

$$\mathcal{T} = \frac{1}{\sigma_V^2}\int_0^\infty \overline{\mathbf{v}'(t)\mathbf{v}'(t+\tau)}\mathrm{d}\tau \tag{6}$$

The dispersion of fluid particles injected at a source point at time $t = 0$ is given by Taylor's theorem (7–9):

$$\frac{d\overline{\mathbf{r}^2(t)}}{dt} = 2\overline{\mathbf{r}(t)\mathbf{v}(t)} = 2\int_0^t \overline{\mathbf{v}(t')\,\mathbf{v}(t)}\mathrm{d}t'. \tag{7}$$

Applied to equation (5) at time $t = x/\overline{v}$, the transverse spread is (7, 8, 10, 11):

$$\sigma_R^2 = \frac{2}{3}\sigma_V^2\mathcal{T}^2\left[\exp\left(-\frac{x}{\overline{v}\mathcal{T}}\right) + \frac{x}{\overline{v}\mathcal{T}} - 1\right] \tag{8}$$

At short and long distances compared to $\overline{v}\mathcal{T}$, σ_R can be rewritten as the mean square displacement at time $t = x/\overline{v}$ of a diffusive process with an effective diffusion coefficient D:

$$\sigma_R^2 \sim D\frac{x}{\overline{v}} \tag{9}$$

The effective diffusion coefficient is constant at long distances $x \gg \overline{v}\mathcal{T}$, as expected for an uncorrelated random walk of

velocity σ_V and mean free path $\sigma_V \mathcal{T}$:

$$D \sim \sigma_V^2 \mathcal{T} \tag{10}$$

At short distances $x \ll \overline{v}\mathcal{T}$, particle motion is correlated so that the mean free path is rather the distance traveled σ_R:

$$D \sim \sigma_V \sigma_R \tag{11}$$

This gives the two scalings reported in the article.

Unlike passive scalars, particles are subjected to gravity and inertia. The equation of motion of a single particle reads (12)

$$\frac{\mathrm{d}}{\mathrm{d}t}\mathbf{r} = \mathbf{v}, \quad \frac{\mathrm{d}}{\mathrm{d}t}\mathbf{v} = -\frac{1}{\tau_S}(\mathbf{v} - \mathbf{u}[\mathbf{r}]) + \left(1 - \frac{\rho_f}{\rho_p}\right)\mathbf{g} \tag{12}$$

where $\mathbf{u} = \overline{\mathbf{u}} + \mathbf{u}'$ is the fluid velocity and $\tau_S = \rho_p d^2/18\eta$ the Stokes times, which is the particle response time to a change in the fluid velocity given by the Stokes force $-3\pi\eta d(\mathbf{v} - \mathbf{u})$. $(1 - \rho_f/\rho_p)\,\mathbf{g}$ is a buoyancy term. In the absence of turbulence, particles fall with a velocity

$$\mathbf{v}_{\text{fall}} = \frac{(\rho_p - \rho_f)\mathbf{g}d^2}{18\eta}. \tag{13}$$

If the fluid presents a constant mean velocity, particles move on average at a velocity $\overline{\mathbf{v}} = \overline{\mathbf{u}} + \mathbf{v}_{\text{fall}}$. We consider again for simplicity that the fluid velocity correlation function (but not the particle velocity correlation function $\overline{\mathbf{v}'(t)\mathbf{v}'(t+\tau)}$) decays exponentially over a time $\mathcal{T}$:

$$\overline{\mathbf{u}'(t)\mathbf{u}'(t+\tau)} = \sigma_U^2 \exp(-\tau/\mathcal{T}) \tag{14}$$

The aerosol phase forms when turbulent velocity fluctuations are large enough to counteract particle settling (13) i.e. when $\sigma_U > v_{\text{fall}}$.

The fluctuating velocity obeys:

$$\mathbf{v}'(t) = \frac{1}{\tau_S}\int_{-\infty}^{t} \mathbf{u}'(t') \exp\left(-\frac{t - t'}{\tau_S}\right)\mathrm{d}t' \tag{15}$$

The particle velocity correlation function is the low-passed fluid velocity correlation:

$$\overline{\mathbf{v}'(t)\mathbf{v}'(t+\tau)} = \frac{1}{2\tau_S}\int_{-\infty}^{\tau} \overline{\mathbf{u}'(t_2)\mathbf{u}'(t_2+t_1)} \exp\left(-\frac{\tau - t_1}{\tau_S}\right)\mathrm{d}t_1 \tag{16}$$

Performing the Reynolds decomposition $\mathbf{v} = \overline{\mathbf{v}} + \mathbf{v}'$ and using the Fourier transform of the fluid velocity autocorrelation function, we find:

$$\overline{\mathbf{v}'(t)\mathbf{v}'(t+\tau)} = \frac{\sigma_U^2}{1 - \frac{\tau_S^2}{\mathcal{T}^2}}\left(\exp\left(-\tau/\mathcal{T}\right) - \frac{\tau_S}{\mathcal{T}}\exp\left(-\tau/\tau_S\right)\right) \tag{17}$$

Inertia acts as a low-pass filter of the fluid velocity (14). The dimensionless ratio $\mathrm{St} = \tau_S/\mathcal{T}$ is called the Stokes number and characterizes the relative influence of particle inertia and hydrodynamic drag. If the Stokes number St is much smaller than 1, particles presents a negligible inertia and the particle velocity correlation function reduces to the fluid velocity correlation function $\overline{\mathbf{v}'(t)\mathbf{v}'(t+\tau)} = \overline{\mathbf{u}'(t)\mathbf{u}'(t+\tau)}$. Conversely, in the limit where the Stokes number St is much larger than 1, the correlation function decays exponentially as $\sigma_U^2 \mathrm{St} \exp\left(-\tau/\tau_S\right)$.

Turbulent dispersion: Reynolds-averaged Eulerian approach

In the continuum approximation, we introduce the concentration $C(\mathbf{r}, t) = \overline{C}(\mathbf{r}, t) + C'(\mathbf{r}, t)$ of particles and its flux $\mathbf{j}$. The conservation equation reads:

$$\frac{\partial C}{\partial t} + \nabla \cdot \mathbf{j} = 0 \tag{18}$$

Transport by the the average flow leads to a flux equal to the concentration times velocity $\mathbf{j} = C\mathbf{v}$. Consider now thermal diffusion induced by random microscopic velocity fluctuations at $\mathbf{v} = \mathbf{0}$. Would the concentration be homogeneous i.e. constant in space, the diffusive flux would vanish. At leading order, random exchanges between neighboring layers of fluid lead to a flux proportional to the gradient of concentration, oriented from high to low concentration. The phenomenological relation is known as Fick's law:

$$\mathbf{j} = -D_m \nabla C \tag{19}$$

and the molecular diffusion coefficient D_m is assumed to be independent of C, which must be true in the dilute limit $C \to 0$. Plugging this relation into the continuity equation, we get the linear diffusion equation:

$$\frac{\partial C}{\partial t} = D_m \nabla^2 C \tag{20}$$

With turbulence, the relevant transport equation is obtained by Reynolds averaging the concentration conservation equation:

$$\frac{\partial \overline{C}}{\partial t} + \nabla \cdot (\overline{\mathbf{v}}\overline{C}) = \nabla \cdot \left(D_m \nabla \overline{C} - \overline{\mathbf{v}'C'}\right) \tag{21}$$

For the reasons invoked for molecular diffusion, the flux $\overline{\mathbf{v}'C'}$ can be expressed using a gradient diffusion assumption:

$$\overline{\mathbf{v}'C'} = -D_t \nabla \overline{C} \tag{22}$$

where D_t is the turbulent diffusion coefficient, also called eddy diffusivity. The averaged concentration therefore obeys:

$$\frac{\partial \overline{C}}{\partial t} + \nabla \cdot (\overline{\mathbf{v}}\overline{C}) = \nabla \cdot \left(D \nabla \overline{C}\right) \tag{23}$$

where $D = D_m + D_t$ is the effective diffusion coefficient. In the Lagrangian approach, we have introduced the decorrelation time $\mathcal{T}$. For the Eulerian framework, we must introduce the inertial length scale $\mathcal{L}$; above this space length, fluid velocities are uncorrelated.

For a point source at the origin in an average flow along the x direction in cylindrical (r, θ, x) coordinates and neglecting longitudinal diffusion, the average concentration $\overline{C}$ obeys a convection-diffusion equation which reduces in the steady state to:

$$\overline{v}\frac{\partial \overline{C}}{\partial x} = r^{-1}\frac{\partial}{\partial r}\left(r\ D\ \frac{\partial \overline{C}}{\partial r}\right) \tag{24}$$

This equation has the same structure as the diffusion equation, except that time is replaced by the space coordinate x. In the regime described by Taylor, the turbulent diffusion coefficient D does not depend on r, the equation admits an exact solution:

$$\overline{C} = \frac{q_e C_e}{\pi \sigma_R^2 \overline{v}} \exp\left(-\frac{r^2}{2\sigma_R^2}\right) \tag{25}$$

where the multiplicative factor is obtained by identifying the mass flow rate across any section to the source injection rate

$q_e C_e$. The dispersion radius σ_R obeys the equation:

$$\bar{v}\,\frac{\mathrm{d}\sigma_R^2}{\mathrm{d}x} = 2D \tag{26}$$

We now consider the opposite limit where there is no mean flow at all, but convective plumes creating turbulent mixing. For simplicity, we can assume that dispersion is homogeneous and isotropic and write the diffusion equation in spherical coordinates:

$$\frac{\partial \overline{C}}{\partial t} = r^{-2}\frac{\partial}{\partial r}\left(r^2\, D\,\frac{\partial \overline{C}}{\partial r}\right) \tag{27}$$

Again, considering a constant source of flow rate q_e and concentration C_e, a steady state solution gradually appears, which obeys (15):

$$\frac{\mathrm{d}\overline{C}}{\mathrm{d}r} = -\frac{q_e C_e}{4\pi r^2\, D} \tag{28}$$

At large scale, $D \sim \sigma_V \mathcal{L}$ can be considered as constant so that $\overline{C}$ decreases as r^{-1}. In the intermediate range of scales, using again the ballistic approximation $D \sim \sigma_V r$, $\overline{C}$ decreases as r^{-2}. The scaling laws derived before therefore still hold, but up to a geometrically determined multiplicative constant α.

Droplet production

Coughing, sneezing, singing, speaking, laughing or breathing produce droplets of mucosalivary fluid in two range of sizes. Droplets above 100 μm are produced by fragmentation of a liquid sheet formed at the upper end of the respiratory tract (i.e. for sneeze) or from filaments between the lips (i.e. plosives consonants when speaking (16, 17)), with an average around 500 μm (18–20). In the first case, the initial sheet is stretched and get pierced. The liquid accumulates by capillarity retraction in a rim, which destabilizes into ligaments (21, 22). The latter form droplets by a capillary instability referred to as the beads-on-a-string (23). In the second case, a film forms between the lips, which destabilizes into filaments, themselves exhibiting the beads-on-a-string instability.

Droplets below 20 μm form either by bubble bursting events in the lungs alveoli or by turbulent destabilization of liquid films covering the lower and upper airways. Between 20 μm and 100 μm, almost no droplets form, indicating well separated mechanisms. For each class of droplets, the distribution around the average has been fitted either by a log-normal distribution, following the idea of a break-up cascade, or by a Gamma distribution, based on the idea that the blobs that make up a ligament exhibit an aggregation process before breaking up (24). The average droplet size, around 4 μm results from an interplay between the fluid film thickness, the turbulent stress and the surface tension. Further fragmentation of these droplets can occur in the tract constrictions where air flows at large velocity. Pulmonary surfactant helps reduce the droplet size, and contributes to prevent accumulation of fluid in airways. The main entry zone is through the nasal epithelium for viral strains before Omicron and more specifically a subset of cells of the nasal epithelium expressing both the ACE2 receptor and the TMPRSS2 protease, and the throat epithelium for Omicron, due to a weaker dependency to the TMPRSS2 protease. Other entry zones exist as well as different receptors and proteases (25). In first approximation, they can be considered as minor routes in the dissemination of the epidemic and in the risk of infection. The emission of mucus droplets containing viral particles in the nasal cavity is dominant, but has not been much investigated so far (26, 27).

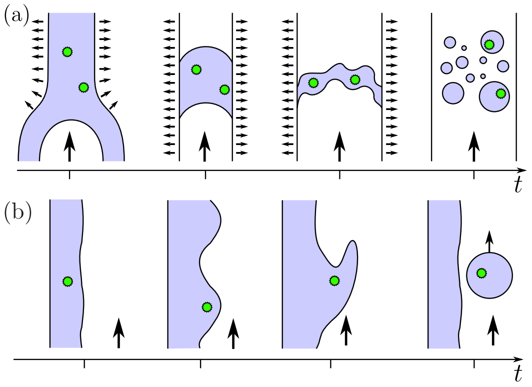

Fig. S3. (a) Sketch of the aerosol generation mechanism inside the bronchioles. The walls are elastic and collapse during exhalation; when air flows back into the bronchioles, it spreads them apart, leading to the formation of a fluid film which then bursts into submicronic droplets (28–32). (b) Sketch of the aerosol generation mechanism inside the upper respiratory tract. Shear destabilizes the respiratory fluid layer lining the walls (30, 33, 34), and droplets detach from the waves. In both cases, if virions are present, they get entrained when the film forms and are found in the subsequent aerosol droplets.

Evaporation process and virus inactivation

We discuss here a basic model of evaporation of droplets, before efflorescence, in humid air. The evaporation of liquid droplets in the air is controlled by the ambient relative humidity RH. Mass transport of water molecules from the droplets to the surrounding air is diffusive; as the drops evaporate, they release latent evaporation heat, which is also conducted away. This cools down the droplets, which in turns lowers the saturation pressure in the immediate surrounding of the drop, slowing down evaporation. Due to this coupled transport, a drop of initial radius a_0 shrinks to a radius $a(t)$ as (35, 36)

$$a^2(t) = a_0^2 - 2D_{\mathrm{eff}}\,(1 - \mathrm{RH})\,t \tag{29}$$

where $D_{\mathrm{eff}} = 1.3\,10^{-10}$ m^2/s is an effective diffusion coefficient taking into account both diffusive transport of mass and its slowdown due to evaporative cooling. Evaporation takes places at the surface of the drop, which explains the linear behavior of a^2 with t. Since the drops are small, evaporation is very fast compared to the time they spend aloft: a 4 μm drop at 70 % RH completely evaporates in 0.2 s, while it takes 20 min to fall a distance of 2 m under its own weight.

The classical picture (37) considers evaporating droplets as independent. This is true for small droplets dispersed inside a room, but not of droplets inside a cough or sneeze spray. In that case, RH is roughly uniform and close to 100 % inside the aerosol jet, meaning that no evaporation takes places except at the spray boundaries (38, 39). This makes these drops extremely long-lived, up to a hundred times the isolated drop lifetime (36, 40, 41).

However, virus-laden respiratory droplets do not vanish as they contain viral particles and are not composed of pure water. The mucosalivary fluid is a dilute solution of surfactants, proteins and electrolytes, initially composed of $\sim$ 99 % water in volume. The solutes stabilize drops at a finite radius a_{eq}, at which they still contain water (42):

$$a_{\mathrm{eq}} = \left(\frac{M_w}{\rho_w}\sum_i \frac{\nu_i c_i}{M_i}\right)^{1/3} \frac{a_0}{(1-\mathrm{RH})^{1/3}} \tag{30}$$

The sum is done over all solutes i. c_i is the mass concentration of solute i, M_i its molar mass, ν_i its degree of dissociation (2 for NaCl). We model respiratory fluid by a mixture of NaCl and the total average protein content (43, 44): $c_{\text{electrolytes}} = 9\,\text{g/L}$ (physiological NaCl concentration), $c_{\text{proteins}} = 70\,\text{g/L}$, $M_{\text{proteins}} = 70\,\text{kg/mol}$. This gives $a_{\text{eq}} \approx 10^{-1} a_0\ (1 - \text{RH})^{-1/3}$: typically, at medium relative humidity, aerosol drops formed at 5 μm remain at 1 μm, which is significantly larger than the virus itself. By stabilizing the droplet at a finite radius, solutes reduce the evaporation time to

$$t_{\text{ev}} = \frac{a_0^2}{2D_{\text{eff}}(1 - \text{RH})}\left(1 - \left(\frac{a_{\text{eq}}}{a_0}\right)^2\right) \tag{31}$$

The solute effect on the evaporation time is small at low RH since the drop shrinks by a lot; at 99 % RH, a 4 μm drop has its evaporation time increased by 80 %.

In aerosol droplets at equilibrium, virions are gradually degraded by the damage done by antiviral proteins in saliva (45) and by UV-B radiation in sunlight or by UV-C. The inactivation rate of enveloped, airborne viruses increases (46–49) with RH: this suggests that virions can associate with proteins which protects them both from dessication and antivirals (44). Another possible effect of ambient conditions results the intrinsic temperature preference of the coronavirus spike protein (50), as the temperature in the nose depends on both temperature and humidity.

Masks and respirators

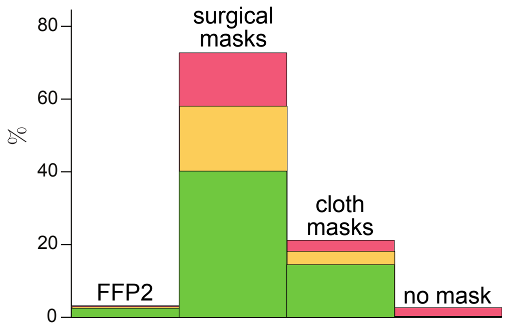

Fig. S4. Mask wearing in public spaces of the Paris suburban region (Ile-de-France) where face masks are mandatory, mid April 2021. $N = 1708$. Green: correct fitting. Orange: centimeter-scale leakage along the nose or the cheeks. Red: No fitting at all.

λ_i and λ_e are respectively the inhalation and exhalation filtration factor of the face coverings worn by the two people. Filtration factors are determined by the material properties, the fitness of the face coverings and the respiratory activity at play. Cloth masks have a material filtration efficiency of 20%–40% (51–57), tightly woven material being more efficient. Surgical masks have a 50%–95% material filtration efficiency (51, 53, 58, 59), and N95/FFP2 respirators 95%–99% (53–55, 60). Inhalation (i.e. exposure reduction) and exhalation (i.e. aerosol source control) are not symmetrical as they induce different airflows around the face covering. Moreover, the flow is different for instance for coughs and tidal breathing, which is associated with a 20 times smaller typical flow rate (61). Surgical masks block 56%–86% of cough aerosols and 42%–91% of exhaled aerosols (58, 62–64); N95 respirators block 73%–99% of cough aerosols (58, 63, 64) and 95%–99% of exhaled aerosols (58, 64). Efficiencies for exposure reduction are roughly similar at the most penetrating particle size in well-mixed conditions (51, 65). Filtration efficiency strongly depends on proper mask fit: cloth and surgical masks tend to be much looser fitted than respirators, thereby greatly reducing their filtration factor compared to their material efficiency (66). Improved surgical mask fit by double masking or knotting and tucking can bring their filtration efficiency up to 60%–99% (62, 67–69). We take as an average $\lambda = \sqrt{\lambda_i \lambda_e}$ for both inhalation and exhalation. $\lambda = 1$ when no mask is worn; $\lambda = 0.70$ for cloth masks (30% efficiency); $\lambda = 0.28$ for well-fitted surgical masks (72% efficiency); $\lambda = 0.10$ for N95 respirators (90% efficiency). For respirators, fit checks are needed to reach high efficiencies, protective enough for workplace aerosol exposure. However, most untrained users achieve on their own 90% and higher filtration, making respirators a valuable choice for the general public (70, 71).

We have performed a quantitative study of face mask wearing in public spaces around Paris. Investigators pair up in a public place and observe the people around them without taking notes, keeping their count. One of the two investigators is familiar with the public place and the other not. They decide on the type of mask they wear and the most appropriate memorization technique, after a reconnaissance visit. Every 10 people, they discreetly write down the quantitative measurements on the type of mask they wear and on the mask fitting. Every twenty minutes, in a calm environment, without witnesses, they discuss the social characteristics of the place under study, at this time of day and this day of the week, in order to prevent perception biases. Ideally, the investigators fill, for each person, the following categories: gender, age, occupational characteristics, visible religious attributes, spoken language, alone or accompanied, behavior. Special attention is paid to the difference between externally imposed rules vs internalized rules, when noting the behaviors in the presence or not of a control (72–74).

Social aspects will be discussed in a separate paper. Here, it is used to ensure that face mask wearing statistics reflect social activities at different times of the week, in the Paris region. Figure S4 shows the resulting histogram. Only indoor public spaces where masks are mandatory have been included. FFP2 respirators represent 3 % only of face barriers and correctly worn protective coverings (surgical or FFP2) only 42 %. We can estimate that the mean filtration factor $\lambda_i \lambda_e$ is around 0.25 only, which gives a dose reduction by a factor of 4 in French public spaces where masks are mandatory. As even minute differences in respirator fit can lead to large variations in filtration, this visual method of estimating mask fit could lead to overestimating the average filtration efficiency. In Georgia, USA during November-December 2020, mask wearing for students and staff members was associated with a 2.7-fold reduction in COVID-19 cases (75). In the UK, between February and April 2021, schoolchildren aged 10-14 did not wear masks while those aged 15-19 did. Figure S5 compares the cases in these two populations. The reproduction rate that can be attributed to schools is roughly halved by masks, which gives a mean filtration factor $\lambda^2 = 0.5$. We have observed that young people poorly fit their masks, which could explain the discrepancy with the expected filtration factor. Another explanation could be that an important part of the transmission takes places during unmasked lunch time.

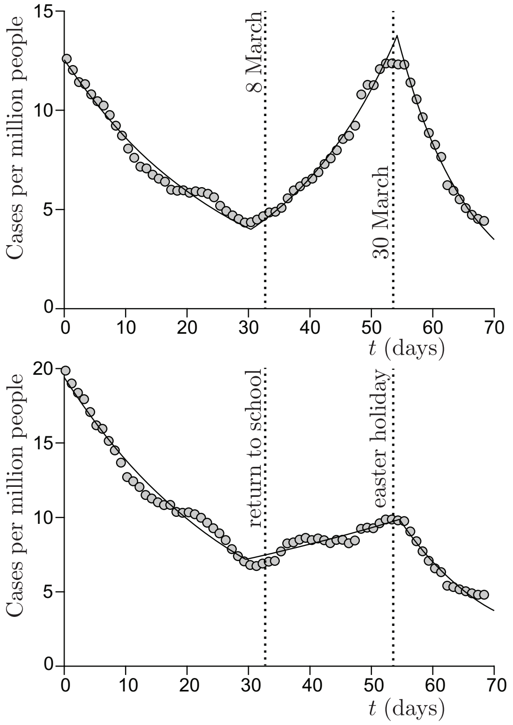

Fig. S5. Cases per million people in the United Kingdom, from 1 February to 12 April 2021. Top panel: schoolchildren aged 10-14, with no mandatory masks. The best fit by an exponential provides the reproduction number: $R = 1.34 \pm 0.04$ during the school period vs $R = 0.81 \pm 0.03$ before and $R = 0.70 \pm 0.03$ after. Bottom panel: schoolchildren aged 15-19, with mandatory masks. The best fit by an exponential provides the reproduction number: $R = 1.07 \pm 0.04$ during the school period vs $R = 0.82 \pm 0.03$ before and $R = 0.70 \pm 0.03$ after.

Risk reduction techniques

The transmission risk outdoors, without masks, is real at short distances and for long periods of time. Figure S7 shows the risk as a function of distance. The risk is above 1 at low wind speeds below 2.5 m. Static crowds without masks must therefore be avoided. It is important for people to learn how to take into account the wind strength and direction for static outdoors activities, in particular if they sing, eat, or drink for a long duration. As the risk outdoors is entirely at short range, large fans may be used to reduce the risk of a bar terrace or static queue in front of a shop (figure S6 panel b). The more these fans induce turbulent fluctuations, rather than an average flow, the better they are. They must be oriented upwards to change the wake direction. For outdoor dance floors, injection of air at high flow rate, say, $1 - 10$ m^3/hour/person may be sufficient to reduce the risk.

The absence of masks when eating and drinking poses a specific problem of aerosol risk reduction. In particular, collective catering facilities are amongst the most important places of high transmission risk. It is possible to use HEPA filtered air purifiers arranged to provide air free of viral particles and suck out stale air (76) (Figure S6 a, e).

Disposable respiratory personal protection equipment is expensive, but various protocols involving UV-C, heat and hydrogen peroxide vapor have been designed to extend its lifetime cycle and use, and could be considered for the general public (77, 78).

References

1. Risken H, Frank T 1989. *The Fokker-Planck Equation - Methods of Solution and Applications*. No. 18 in Springer Series in Synergetics. Springer Berlin Heidelberg, second ed.
2. Lesieur M 2008. *Turbulence in Fluids*. No. 84 in Fluid Mechanics and Its Applications. Springer, Dordrecht, 4. rev. and enlarged ed ed. ISBN 978-1-4020-6434-0 978-1-4020-6435-7.
3. Batchelor GK 1949. Diffusion in a Field of Homogeneous Turbulence. I. Eulerian Analysis. Australian Journal of Chemistry, 2(4):437–450.
4. Batchelor GK 1950. The application of the similarity theory of turbulence to atmospheric diffusion. Quarterly Journal of the Royal Meteorological Society, 76(328):133–146.
5. Batchelor GK 1952. Diffusion in a field of homogeneous turbulence: II. The relative motion of particles. Mathematical Proceedings of the Cambridge Philosophical Society, 48(2):345–362.
6. Sawford BL 1991. Reynolds number effects in Lagrangian stochastic models of turbulent dispersion. Physics of Fluids A: Fluid Dynamics, 3(6):1577–1586.
7. Sawford B 2001. Turbulent Relative Dispersion. Annual Review of Fluid Mechanics, 33(1):289–317.
8. Salazar JP, Collins LR 2009. Two-Particle Dispersion in Isotropic Turbulent Flows. Annual Review of Fluid Mechanics, 41(1):405–432.
9. Taylor GI 1922. Diffusion by Continuous Movements. Proceedings of the London Mathematical Society, s2-20(1):196–212.
10. Du S, *et al.* 1995. Estimation of the Kolmogorov constant for the Lagrangian structure function, using a second-order Lagrangian model of grid turbulence. Physics of Fluids, 7(12):3083–3090.
11. Poydenot F, *et al.* 2021. Turbulent dispersion of breath by the wind. arXiv:211206501 [physics].
12. Maxey MR, Riley JJ 1983. Equation of motion for a small rigid sphere in a nonuniform flow. The Physics of Fluids, 26(4):883–889.
13. Friedlander SK 2000. *Smoke, Dust, and Haze*, vol. 198. Oxford University Press, New York.
14. Bec J, *et al.* 2010. Turbulent pair dispersion of inertial particles. Journal of Fluid Mechanics, 645:497–528.
15. Cheng KC, *et al.* 2011. Modeling Exposure Close to Air Pollution Sources in Naturally Ventilated Residences: Association of Turbulent Diffusion Coefficient with Air Change Rate. Environmental Science & Technology, 45(9):4016–4022.
16. Abkarian M, *et al.* 2020. Speech can produce jet-like transport relevant to asymptomatic spreading of virus. Proceedings of the National Academy of Sciences, 117(41):25237–25245.
17. Abkarian M, Stone HA 2020. Stretching and break-up of saliva filaments during speech: A route for pathogen aerosolization and its potential mitigation. Physical Review Fluids, 5(10):102301.
18. Morawska L, *et al.* 2009. Size distribution and sites of origin of droplets expelled from the human respiratory tract during expiratory activities. Journal of Aerosol Science, 40(3):256–269.
19. Johnson GR, *et al.* 2011. Modality of human expired aerosol size distributions. Journal of Aerosol Science, 42(12):839–851.

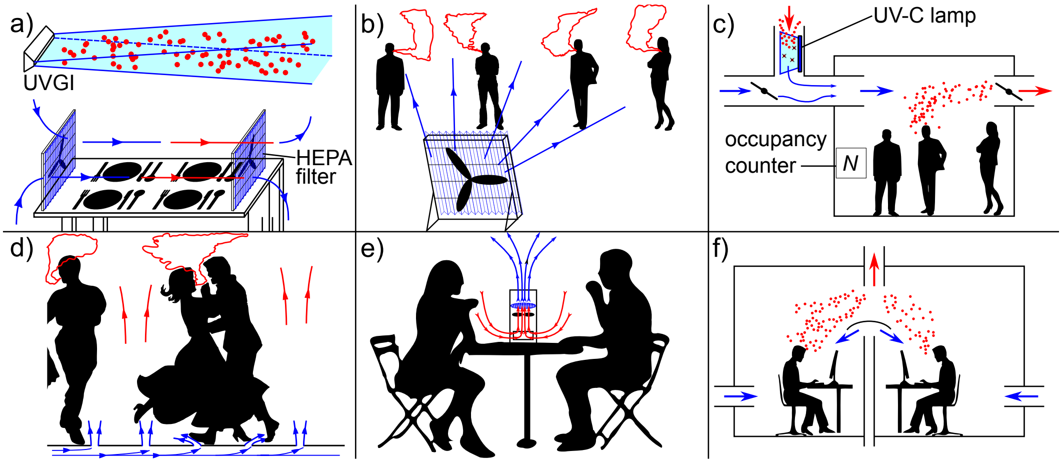

Fig. S6. Hydrodynamic solutions to mitigate short-range transmission by dispersing aerosols away from individuals. (a) Fans force the circulation of air through HEPA filters at a cafeteria table, with an Upper-Room Ultraviolet Germicidal Irradiation (UVGI). (b) A large fan with a filter is slightly tilted upward and aimed at a line of static people waiting. The cone of aerosols they emit cannot reach anyone in the line. (c) Ventilation controlled by the occupancy number, to reduce energy consumption. Recycled air inside ventilation ducts is decontaminated by UV-C light. (d) An air flow inside the floor of a club pushes air up through small holes, preventing aerosols from spreading laterally and dispersing them towards the ceiling where the ventilation system can remove them. (e) A fan at a café table pulls air, filters it and expels it upwards. (f) Fresh air is injected at the bottom of the room and supplied where needed to create air flows that protect each occupant of the room individually (76).

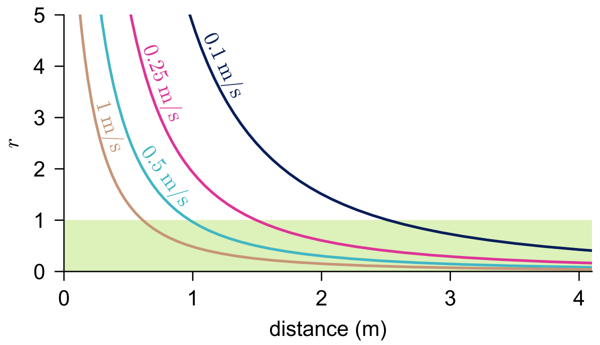

Fig. S7. Transmission risk r for Omicron BA.1 ($\bar{h} = 2800$) as a function of the distance between people inside the public space, for the observed mask wearing shown in Figure S4 ($\lambda^2 = 0.2$) and different wind speeds. Green area: acceptable risk, ie. $r < 1$.

20. Bourouiba L 2021. The Fluid Dynamics of Disease Transmission. Annual Review of Fluid Mechanics, 53(1):473–508.
21. Kooij S, *et al.* 2018. What Determines the Drop Size in Sprays? Physical Review X, 8(3):031019.
22. Bourouiba L, Dehandschoewercker E, Bush JM 2014. Violent expiratory events: on coughing and sneezing. Journal of Fluid Mechanics, 745:537–563.
23. Scharfman BE, *et al.* 2016. Visualization of sneeze ejecta: steps of fluid fragmentation leading to respiratory droplets. Experiments in Fluids, 57(2):24.
24. Villermaux E 2007. Fragmentation. Annual Review of Fluid Mechanics, 39(1):419–446.
25. Singh M, Bansal V, Feschotte C 2020. A Single-Cell RNA Expression Map of Human Coronavirus Entry Factors. Cell Reports, 32(12):108175.
26. Li X, *et al.* 2021. Detecting SARS-CoV-2 in the Breath of COVID-19 Patients. Frontiers in Medicine, 8.
27. Ma J, *et al.* 2021. Coronavirus Disease 2019 Patients in Earlier Stages Exhaled Millions of Severe Acute Respiratory Syndrome Coronavirus 2 Per Hour. Clinical Infectious Diseases, 72(10):e652–e654.
28. Malashenko A, Tsuda A, Haber S 2009. Propagation and Breakup of Liquid Menisci and Aerosol Generation in Small Airways. Journal of Aerosol Medicine and Pulmonary Drug Delivery, 22(4):341–353.
29. Johnson GR, Morawska L 2009. The Mechanism of Breath Aerosol Formation. Journal of Aerosol Medicine and Pulmonary Drug Delivery, 22(3):229–237.
30. Mittal R, Ni R, Seo JH 2020. The flow physics of COVID-19. Journal of Fluid Mechanics, 894:F2.
31. Halpern D, Grotberg JB 2003. Nonlinear saturation of the Rayleigh instability due to oscillatory flow in a liquid-lined tube. Journal of Fluid Mechanics, 492:251–270.
32. Haslbeck K, *et al.* 2010. Submicron droplet formation in the human lung. Journal of Aerosol Science, 41(5):429–438.
33. Kataoka I, Ishii M, Mishima K 1983. Generation and Size Distribution of Droplet in Annular Two-Phase Flow. Journal of Fluids Engineering, 105(2):230–238.
34. Moriarty JA, Grotberg JB 1999. Flow-induced instabilities of a mucus–serous bilayer. Journal of Fluid Mechanics, 397:1–22.
35. Pruppacher H, Klett J 2010. *Microphysics of Clouds and Precipitation*, vol. 18 of *Atmospheric and Oceanographic Sciences Library*. Springer Netherlands, Dordrecht. ISBN 978-0-7923-4211-3 978-0-306-48100-0.
36. Smith SH, *et al.* 2020. Aerosol persistence in relation to possible transmission of SARS-CoV-2. Physics of Fluids, 32(10):107108.
37. Wells WF 1934. On air-borne infection. Study II. Droplets and droplet nuclei. American Journal of Hygiene, 20:611–18.
38. Villermaux E, *et al.* 2017. Fine structure of the vapor field in evaporating dense sprays. Physical Review Fluids, 2(7):074501.

39. Ng CS, *et al.* 2021. Growth of respiratory droplets in cold and humid air. Physical Review Fluids, 6(5):054303.
40. Chong KL, *et al.* 2021. Extended Lifetime of Respiratory Droplets in a Turbulent Vapor Puff and Its Implications on Airborne Disease Transmission. Physical Review Letters, 126(3):034502.
41. de Oliveira PM, *et al.* 2021. Evolution of spray and aerosol from respiratory releases: theoretical estimates for insight on viral transmission. Proceedings of the Royal Society A: Mathematical, Physical and Engineering Sciences, 477(2245):20200584.
42. Mikhailov E, *et al.* 2004. Interaction of aerosol particles composed of protein and salts with water vapor: hygroscopic growth and microstructural rearrangement. Atmos Chem Phys, 4:323—350.
43. Nicas M, Nazaroff WW, Hubbard A 2005. Toward Understanding the Risk of Secondary Airborne Infection: Emission of Respirable Pathogens. Journal of Occupational and Environmental Hygiene, 2(3):143–154.
44. Vejerano EP, Marr LC 2018. Physico-chemical characteristics of evaporating respiratory fluid droplets. Journal of The Royal Society Interface, 15(139):20170939.
45. Malamud D, *et al.* 2011. Antiviral Activities in Human Saliva. Advances in Dental Research, 23(1):34–37.
46. Yang W, Marr LC 2011. Dynamics of Airborne Influenza A Viruses Indoors and Dependence on Humidity. PLoS ONE, 6(6):e21481.
47. Tang JW 2009. The effect of environmental parameters on the survival of airborne infectious agents. Journal of The Royal Society Interface, 6:S737–S746.
48. Chan KH, *et al.* 2011. The effects of temperature and relative humidity on the viability of the SARS coronavirus. Advances in virology, 2011.
49. Smither SJ, *et al.* 2020. Experimental aerosol survival of SARS-CoV-2 in artificial saliva and tissue culture media at medium and high humidity. Emerging Microbes & Infections, 9(1):1415–1417.
50. Laporte M, *et al.* 2021. The SARS-CoV-2 and other human coronavirus spike proteins are fine-tuned towards temperature and proteases of the human airways. PLOS Pathogens, 17(4):e1009500.
51. Pan J, *et al.* 2021. Inward and outward effectiveness of cloth masks, a surgical mask, and a face shield. Aerosol Science and Technology, 55(6):718–733.
52. Fischer EP, *et al.* 2020. Low-cost measurement of face mask efficacy for filtering expelled droplets during speech. Science Advances, 6(36):eabd3083.
53. Hill WC, Hull MS, MacCuspie RI 2020. Testing of Commercial Masks and Respirators and Cotton Mask Insert Materials using SARS-CoV-2 Virion-Sized Particulates: Comparison of Ideal Aerosol Filtration Efficiency versus Fitted Filtration Efficiency. Nano Letters, 20(10):7642–7647.
54. Zangmeister CD, *et al.* 2020. Filtration Efficiencies of Nanoscale Aerosol by Cloth Mask Materials Used to Slow the Spread of SARS-CoV-2. ACS Nano, 14(7):9188–9200.
55. Rengasamy S, Eimer B, Shaffer RE 2010. Simple Respiratory Protection—Evaluation of the Filtration Performance of Cloth Masks and Common Fabric Materials Against 20–1000 nm Size Particles. The Annals of Occupational Hygiene, 54(7):789–798.
56. Shakya KM, *et al.* 2017. Evaluating the efficacy of cloth facemasks in reducing particulate matter exposure. Journal of Exposure Science & Environmental Epidemiology, 27(3):352–357.
57. Drewnick F, *et al.* 2021. Aerosol filtration efficiency of household materials for homemade face masks: Influence of material properties, particle size, particle electrical charge, face velocity, and leaks. Aerosol Science and Technology, 55(1):63–79.
58. Lindsley WG, *et al.* 2021. A comparison of performance metrics for cloth masks as source control devices for simulated cough and exhalation aerosols. Aerosol Science and Technology, 55(10):1125–1142.
59. Oberg T, Brosseau LM 2008. Surgical mask filter and fit performance. American Journal of Infection Control, 36(4):276–282.
60. Qian Y, *et al.* 1998. Performance of N95 Respirators: Filtration Efficiency for Airborne Microbial and Inert Particles. American Industrial Hygiene Association Journal, 59(2):128–132.
61. Pöhlker ML, *et al.* 2021. Respiratory aerosols and droplets in the transmission of infectious diseases. arXiv:210301188 [physics].
62. Blachere FM, *et al.* 2021. Face mask fit modifications that improve source control performance. medRxiv.
63. Lindsley WG, *et al.* 2021. Efficacy of face masks, neck gaiters and face shields for reducing the expulsion of simulated cough-generated aerosols. Aerosol Science and Technology, 55(4):449–457.
64. Asadi S, *et al.* 2020. Efficacy of masks and face coverings in controlling outward aerosol particle emission from expiratory activities. Scientific Reports, 10(1):15665.
65. Lindsley WG, *et al.* 2021. Efficacy of universal masking for source control and personal protection from simulated cough and exhaled aerosols in a room. Journal of Occupational and Environmental Hygiene, 18(8):409–422.
66. Cappa CD, *et al.* 2021. Expiratory aerosol particle escape from surgical masks due to imperfect sealing. Scientific Reports, 11(1):12110.
67. Sickbert-Bennett EE, *et al.* 2021. Fitted Filtration Efficiency of Double Masking During the COVID-19 Pandemic. JAMA Internal Medicine.
68. Brooks JT, *et al.* 2021. Maximizing Fit for Cloth and Medical Procedure Masks to Improve Performance and Reduce SARS-CoV-2 Transmission and Exposure, 2021. Morbidity and Mortality Weekly Report, 70(7):254–257.
69. Clapp PW, *et al.* 2021. Evaluation of Cloth Masks and Modified Procedure Masks as Personal Protective Equipment for the Public During the COVID-19 Pandemic. JAMA Internal Medicine, 181(4):463–469.
70. Rembialkowski B, Sietsema M, Brosseau L 2017. Impact of time and assisted donning on respirator fit. Journal of Occupational and Environmental Hygiene, 14(9):669–673.
71. Brosseau LM 2010. Fit Testing Respirators for Public Health Medical Emergencies. Journal of Occupational and Environmental Hygiene, 7(11):628–632.
72. Goffman E 1988. *Les moments et leurs hommes.* Seuil : Minuit, Paris. ISBN 978-2-02-009984-4.
73. Hall ET 1990. *The Hidden Dimension.* Anchor Books, New York. ISBN 978-0-385-08476-5.
74. Winkin Y, editor 2000. *La nouvelle communication.* Points. Éd. du Seuil, Paris, éd. rev. et corr ed. ISBN 978-2-02-042784-5.
75. Gettings J 2021. Mask Use and Ventilation Improvements to Reduce COVID-19 Incidence in Elementary Schools — Georgia, November 16–December 11, 2020. MMWR Morbidity and Mortality Weekly Report, 70.

76. Morawska L, *et al.* 2021. A paradigm shift to combat indoor respiratory infection. Science, 372(6543):689–691.
77. CDC 2020. Decontamination and reuse of filtering facepiece respirators. Tech. rep., US Department of Health and Human Services, Atlanta, GA.
78. John AR, *et al.* 2021. Scalable in-hospital decontamination of N95 filtering face-piece respirator with a peracetic acid room disinfection system. Infection Control & Hospital Epidemiology, 42(6):678–687.
